# 1-*O*-Acetylgeopyxin A, a derivative of a fungal metabolite, blocks tetrodotoxin-sensitive voltage-gated sodium, calcium channels and neuronal excitability which correlates with inhibition of neuropathic pain

**DOI:** 10.1186/s13041-020-00616-2

**Published:** 2020-05-11

**Authors:** Yuan Zhou, Song Cai, Kimberly Gomez, E. M. Kithsiri Wijeratne, Yingshi Ji, Shreya S. Bellampalli, Shizhen Luo, Aubin Moutal, A. A. Leslie Gunatilaka, Rajesh Khanna

**Affiliations:** 1 Department of Clinical Laboratory, the First Hospital of Jilin University, Changchun, 130021 China; 2 Department of Pharmacology, College of Medicine, University of Arizona, 1501 North Campbell Drive, P.O. Box 245050, Tucson, AZ 85724 USA; 3 Southwest Center for Natural Products Research, School of Natural Resources & the Environment, College of Agriculture & Life Sciences, The University of Arizona, Tucson, AZ 85724 USA; 4 Neuroscience Graduate Interdisciplinary Program, College of Medicine, Tucson, AZ 85724 USA; 5 The Center for Innovation in Brain Sciences, The University of Arizona Health Sciences, Tucson, AZ 85724 USA

**Keywords:** 1-*O*-acetylgeopyxin a, Voltage-gated calcium channels, Voltage-gated sodium channels, Tetrodotoxin-sensitive voltage-gated sodium channels, Excitability, Non-opioid pain-relieving therapeutics, HIV-induced sensory neuropathy

## Abstract

Chronic pain can be the result of an underlying disease or condition, medical treatment, inflammation, or injury. The number of persons experiencing this type of pain is substantial, affecting upwards of 50 million adults in the United States. Pharmacotherapy of most of the severe chronic pain patients includes drugs such as gabapentinoids, re-uptake blockers and opioids. Unfortunately, gabapentinoids are not effective in up to two-thirds of this population and although opioids can be initially effective, their long-term use is associated with multiple side effects. Therefore, there is a great need to develop novel non-opioid alternative therapies to relieve chronic pain. For this purpose, we screened a small library of natural products and their derivatives in the search for pharmacological inhibitors of voltage-gated calcium and sodium channels, which are outstanding molecular targets due to their important roles in nociceptive pathways. We discovered that the acetylated derivative of the *ent*-kaurane diterpenoid, geopyxin A, 1-*O*-acetylgeopyxin A, blocks voltage-gated calcium and tetrodotoxin-sensitive voltage-gated sodium channels but not tetrodotoxin-resistant sodium channels in dorsal root ganglion (DRG) neurons. Consistent with inhibition of voltage-gated sodium and calcium channels, 1-*O*-acetylgeopyxin A reduced reduce action potential firing frequency and increased firing threshold (rheobase) in DRG neurons. Finally, we identified the potential of 1-*O*-acetylgeopyxin A to reverse mechanical allodynia in a preclinical rat model of HIV-induced sensory neuropathy. Dual targeting of both sodium and calcium channels may permit block of nociceptor excitability and of release of pro-nociceptive transmitters. Future studies will harness the core structure of geopyxins for the generation of antinociceptive drugs.

## Introduction

According to the International Association for the Study of Pain, pain is an unpleasant sensory and emotional experience associated with real or potential tissue damage, which helps the body to be aware of harmful stimuli. Pain can be classified as acute and chronic. Acute pain plays a protective role. This type of pain is short in duration and disappears with the resolution of the pathological process [1]. However, it can transition into chronic pain. Chronic pain persists for a prolonged period of time and it has been estimated to affect one fifth of the world’s population [2]. Chronic pain is pathological, since it is not a symptom of some disorder, but rather, originates from some disease or from a nervous system injury [3]. Although there are many treatment options available, none are universally endorsed, and many come with counterproductive side effects. An estimated 20% of patients with pain-related diagnoses (including acute and chronic pain) receive an opioid prescription. Opioids are associated with multiple side effects including respiratory depression, tolerance, dependence, hyperalgesia, constipation, and mental fog among others [4]. Due to their inefficiency and side effects they affect the quality of life of patients, thus a reduction in the prescription of opioids to treat pain, has been recommended [4]. Thus, there is a tremendous need to develop novel non-opioid pain-relieving therapeutics to provide alternative treatments to manage neuropathic pain.

In this context, in the search for bioactive and/or novel metabolites of plant- and lichen-associated fungi, we have evaluated a library of ~ 90 natural products isolated from Sonoran Desert plants, and some derivatives of these natural molecules. Of these, geopyxins A–F (1 through 5), a group of *ent-*kaurane diterpenoids encountered in endolichenic fungal strains*, Geopyxis aff. Majalis* and *Geopyxis* sp. AZ0066, and their analogs, were found to be active in cancer cell proliferation/survival and heat-shock induction assays [5]. *Ent-*kauranes are known to exhibit a wide range of bioactivities, with cytotoxic [6–9], anti-inflammatory [9–11], antiangiogenic [12], anti-HIV [13], antioxidant [14, 15], anticholinesterase [14], antityrosinase [15], among other properties reported. However, it has not yet been investigated whether these molecules have a therapeutic potential for pain relief.

We recently reported that betulinic acid, a bioactive fraction of the desert plant *Hyptis emoryi,* is antinociceptive in preclinical models of neuropathic pain via targeting of N-type and T-type voltage-gated calcium (Ca^2+^) channels [16]. Likewise, we identified two plant natural products: hardwickiic acid, isolated from *Salvia wagneriana,* and hautriwaic acid, isolated from *Eremocarpus,* both of which reversed pain behaviors in experimental models of HIV-induced and chemotherapy-induced neuropathies by inhibition of voltage-gated sodium (Na^+^) channels [17]. In another study, we demonstrated that physalin F, a steroidal derivative isolated from *Physalis acutifolia* reversed tactile hypersensitivity in models of paclitaxel-induced peripheral neuropathy and spinal nerve ligation (SNL) via blockade of R-type and N-type voltage-gated Ca^2+^ channels [18].

Here, our initial screening of the natural products, geopyxin A and geopyxin C, and geopyxin A derivatives, 1-*O*-acetylmethylgeopyxin A, methylgeopyxin A, and 1-*O*-acetylgeopyxin A revealed that 1-*O*-acetylgeopyxin A inhibits voltage-gated Ca^2+^ channels. Total Na^+^ currents in isolated DRG sensory neurons were also reduced by 1-*O*-acetylgeopyxin A, which preferentially inhibited tetrodotoxin (TTX)-sensitive Na^+^ currents. Sensory neuron excitability was also blunted by 1-*O*-acetylgeopyxin A. Finally, we identified the potential of 1-*O*-acetylgeopyxin A to reverse chronic pain behavior in a preclinical rat model of HIV-Induced sensory neuropathy.

The results presented in this study illustrate the therapeutic potential for 1-*O*-acetylgeopyxin A management of preclinical HIV-induced neuropathy. Likewise, we identified that both voltage-gated Ca^2+^ and Na^+^ channels are the pharmacological targets of 1-*O*-acetylgeopyxin A. Whilst the exact subtype(s) of Ca^2+^ channel remains to be determined, the study illustrates the utility of targeting both sodium and calcium channels in an effort to develop blockers that simultaneously curb excitability and transmitter release.

## Results

### 1-*O*-acetylgeopyxin A preferentially inhibits voltage-gated Ca^2+^ channels

**Fig. 1:**
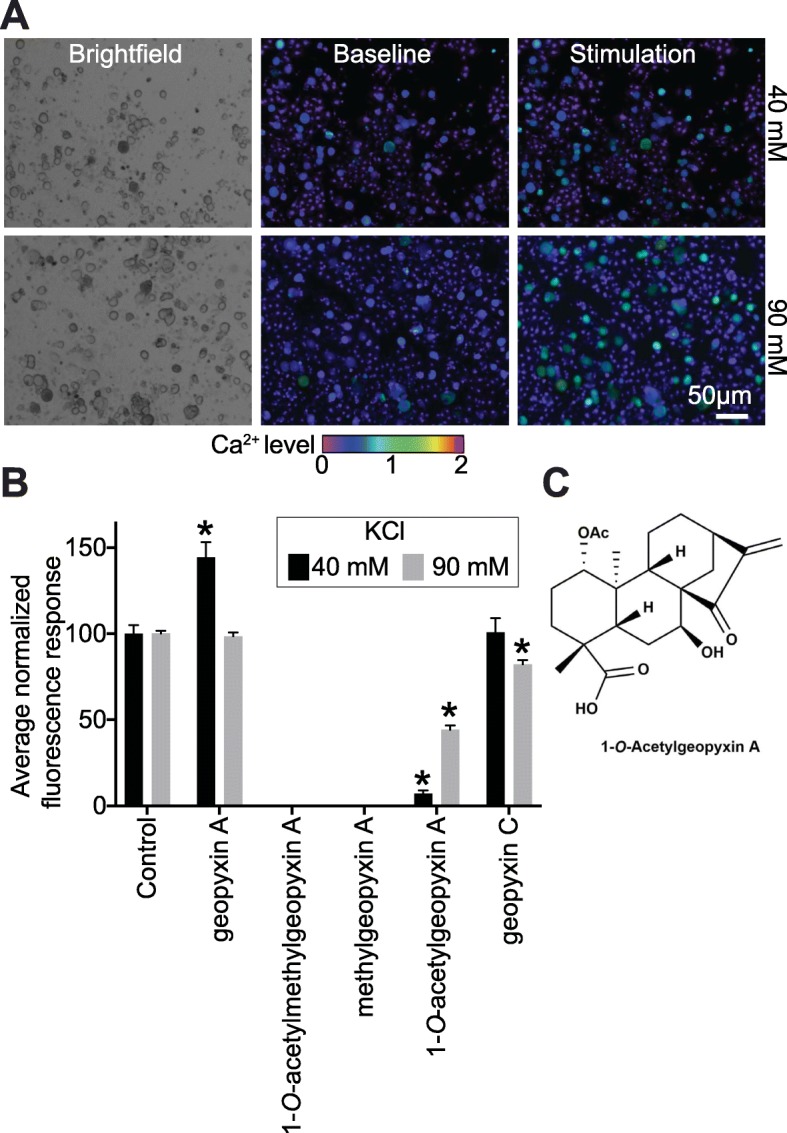
1-*O*-acetylgeopyxin A inhibits calcium influx via voltage-gated calcium channels in sensory neurons. **a** Differential interference contrast (DIC) and pseudocolored fluorescent images of DRG neurons treated with vehicle (1% DMSO), visualized for Fura2-AM before and after stimulations with KCl (40 and 90 mM) during Ca^2+^ imaging. Scale bar is 50 μm. **b** Average responses of sensory neurons incubated overnight with vehicle (control) or 20 μM of geopyxin A, 1-*O*-acetylmethylgeopyxin A, methylgeopyxin A, 1-*O*-acetylgeopyxin A and geopyxin C in response to 40 and 90 mM KCl (to trigger opening of low- and high voltage activated calcium channels) (*n* = 81 to 204 neurons from 3 separate rats). Incubation with 20 μM 1-*O*-acetylmethylgeopyxin A or methylgeopyxin A killed the cells. Asterisk indicate statistical significance compared with cells treated with vehicle (*P < 0.05, one-way ANOVA with Dunnett’s post hoc test). **c** chemical structure of 1-*O*-acetylgeopyxin A. Experimenter was blinded to the treatment condition. Unless otherwise noted, all data is mean ± S.E.M.

To discover new molecules with therapeutic potential for pain relief, we screened a library of natural products for small molecules capable of targeting voltage gated Ca^2+^ channels. This was achieved using Fura2-acetoxymethyl ester (Fura-AM), a ratiometric high-affinity intracellular Ca^2+^ indicator, in rodent sensory dorsal root ganglia (DRG) neurons. First, the neurons were challenged with 40 mM KCl (which changes the membrane voltage to − 32.2 mV – using the Nernst equation) to trigger low voltage-activated (LVA) Ca^2+^ channels, i.e., Cav3.x channels) or 90 mM KCl (which changes the membrane voltage to − 11.4 mV – based on the Nernst equation, to open high-voltage activated (HVA) Ca^2+^ channels, i.e., Cav1.x and Cav2.x) [19] (Fig. 1a). Then, neurons were treated overnight with 20 μM of geopyxin A, 1-*O*-acetylmethylgeopyxin A, methylgeopyxin A, 1-*O*-acetylgeopyxin A and geopyxin C (Fig. 1b). When DRG neurons were treated with 1-*O*-acetylgeopyxin A (Fig. 1c), we noted a significant block of KCl-triggered calcium channel activity when neurons were challenged with 40 and 90 mM of KCl (~ 93 and 56%, respectively, *p < 0.05, one-way ANOVA with Dunnett’s post hoc test) compared to the control (0.1% DMSO). In contrast to the modest degree of inhibition of KCl-triggered calcium influx with geopyxin C when neurons were challenged with 90 mM of KCl (~ 15%, *p < 0.05, one-way ANOVA with Dunnett’s post hoc test), KCl-triggered calcium influx was not affected by geopyxin A compared to DMSO. However, its derivatives, 1-*O*-acetylmethylgeopyxin A and methylgeopyxin A, were highly toxic to DRG neurons (Fig. 1b). These data imply that only 1-*O*-acetylgeopyxin A affects voltage-gated Ca^2+^ channels in sensory neurons.

### 1-*O*-acetylgeopyxin A reduces total calcium currents in DRG sensory neurons

The most potent compound from the primary calcium imaging screening was 1-*O*-acetylgeopyxin A (Fig. 1b), which showed nearly ~ 93 and 56% inhibition of Ca^2+^ channel influx at 20 μM when sensory neurons were depolarized with 40 and 90 mM KCl, respectively. Therefore, we next sought to determine how voltage-gated Ca^2+^ channels were affected by 1-*O*-acetylgeopyxin A using whole-cell patch clamp electrophysiological recordings.

**Fig. 2:**
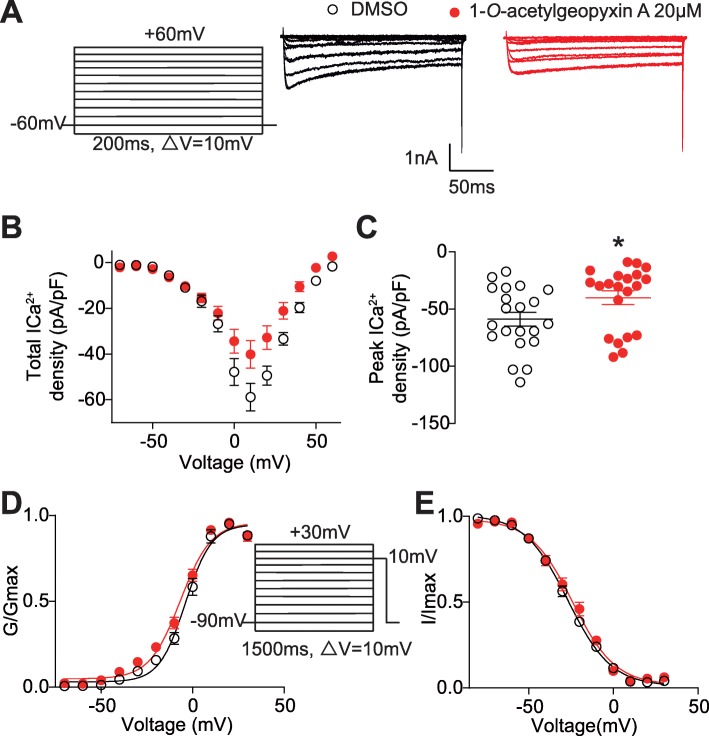
1-*O*-acetylgeopyxin A inhibits total Ca^2+^ currents in dorsal root ganglion (DRG) sensory neurons. **a** Representative traces of total Ca^2+^ currents from DRG sensory neurons treated with 0.1% DMSO (control) or 20 μM 1-*O*-acetylgeopyxin A. Currents were evoked by 200 ms pulse between − 70 and + 60 mV. **b**, **c** Summary of the normalized (pA/pF) total Ca^2+^ current density versus voltage relationship (**b**) and peak total Ca^2+^ current density at + 10 mV (mean ± SEM) (**c**) from DRG sensory neurons treated as indicated. (**d**, **e**) Boltzmann fits for normalized conductance, G/Gmax, voltage relations for voltage dependent activation (**d**) and inactivation (**e**) of sensory neurons treated as indicated. V_1/2_ values for activation and inactivation are presented in Table 1. Asterisks indicate statistical significance compared with cells treated with 0.1% DMSO (*P < 0.05, unpaired two-tailed Student’s t test, *n* = 21 per condition)

Total high voltage-activated (HVA) and low-voltage-activated (LVA)) Ca^2+^ currents were measured from small to medium diameter rat DRG neurons. In order to do so, from a holding potential of − 60 mV, depolarization steps (200 ms step) from − 70 mV to + 60 mV in 10 mV increments were applied (Fig. 2a). Typical traces in Fig. 2a show a family of Ca^2+^ currents recorded from DRG neurons treated overnight with control (0.1% DMSO) (*n* = 21) or 20 μM of 1-*O*-acetylgeopyxin A (n = 21). When compared to the control, 1-*O*-acetylgeopyxin A inhibited total Ca^2+^ current density with a ∼ 31% decrease in peak current density (Fig. 2b, c). The data was normalized according to capacitance of the cell in order to account for the heterogeneity of DRG neuronal populations.

Since this current decrement could be a result of changes in channel gating, we determined the effect of 1-*O*-acetylgeopyxin A on activation and inactivation kinetics of DRG Ca^2+^ currents as described in Methods. After converting the current values to conductance (G), the conductance−voltage relationship was fitted with a Boltzmann equation, and the G value for each neuron was normalized to the maximal value (Gmax) derived from the fit. The G/Gmax−voltage relationship presented in Fig. 2d demonstrates that there was a hyperpolarizing shift of ∼2 mV in the half-maximal activation (V_1/2_) of steady-state. While, a depolarizing shift of ∼3 mV in the V_1/2_ of steady-state inactivation (protocol shown in Fig. 2d inset) in the presence of 1-*O*-acetylgeopyxin A, was observed (Table 1). Thus, these results suggest that 1-*O*-acetylgeopyxin A affects total Ca^2+^ currents.

**Table 1:** Effects of 1-*O*-acetylgeopyxin A on gating properties of Voltage-Gated Channels in DRG Neurons^a^

Calcium Total		
	DMSO	1-*O*-acetylgeopyxin A (20 μM)
Activation		
*V*_*1/2*_ (*P* = 0.0188)	−3.9 ± 0.8 (21)	−6. 7± 0.8 (21)*^b^
*k *(*P* = 0.1966)	7.1 ± 0.7 (21)	8.4 ± 0.7 (21)
Inactivation		
*V*_*1/2 *_(*P* = 0.0363)	−26.8 ± 0.8 (19)	−24.0 ± 1.0 (20)*^b^
*k *(*P* = 0.8775)	−12.8 ± 0.8 (19)	−12.6 ± 1.0 (20)
Sodium		
	DMSO	1-*O*-Acetylgeopyxin A (20 μM)
Activation		
*V*_*1/2*_ (*P* = 0.0001)	−20.1 ± 0.7 (16)	−14.9 ± 0.8 (19)*^b^
*k *(*P* = 0.0013)	4.4 ± 0.6 (16)	7.7 ± 0.7 (19)*^b^
Inactivation		
*V*_*1/2 *_(*P* = 0.0006)	−47.4 ± 1.0 (15)	−40.6 ± 1.4 (18)*^b^
*k *(*P* = 0.1244)	−10.9 ± 0.9 (15)	−13.5 ± 1.3 (18)

^a^Values are means±SEM calculated from fits of the data from the indicated number of individual cells to the Boltzmann equation; *V*_*1/2*_ midpoint potential (mV) for voltage-dependent activation or inactivation; *k*, slope factor. ^b^Significantly different from the value for DMSO (*P < 0.05; Student’s t test)

### 1-*O*-acetylgeopyxin A reduces sodium currents in DRG sensory neurons

**Fig. 3:**
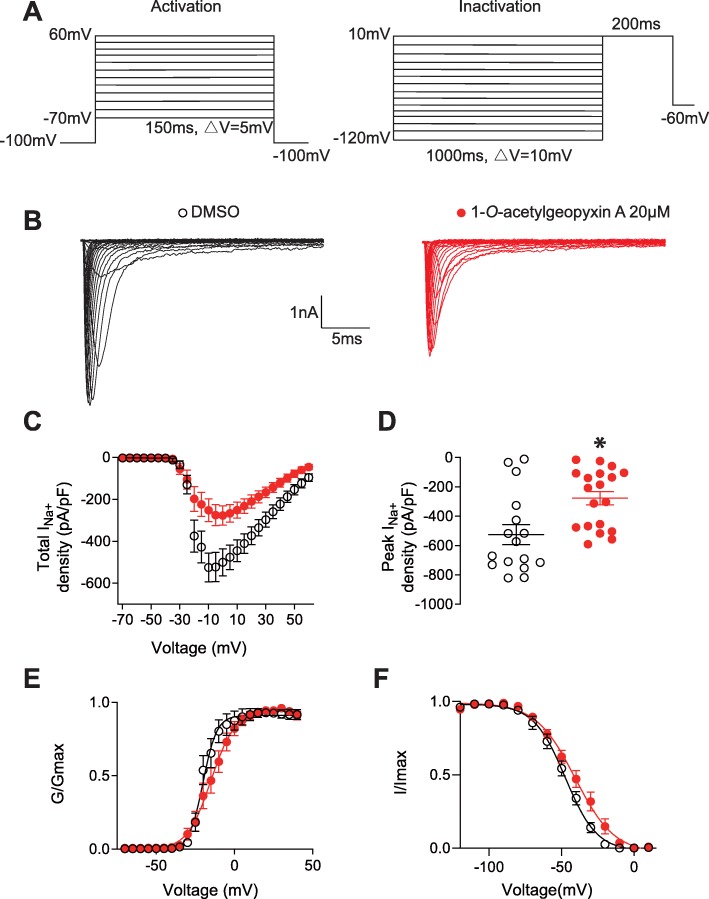
1-*O*-acetylgeopyxin A inhibits total Na^+^ currents in dorsal root ganglion (DRG) neurons. **a** Activation protocol: Currents were evoked by 150 ms pulse between − 70 and + 60 mV (+ 5 mV steps) (left). Inactivation protocol: Cells were subjected to hyperpolarizing/ repolarizing pulses for 1 s between − 120 and 10 mV (+ 10 mV steps), followed by a 0-mV test pulse for 200 ms (right). **b** Representative traces of Na^+^ currents from DRG sensory neurons treated with 0.1% DMSO (control) or 20 μM 1-*O*-acetylgeopyxin A. **c**, **d** Summary (± SEM) of the normalized (pA/pF) sodium current density versus voltage relationship (**c**) and peak Na^+^ current density at − 10 mV (mean ± SEM) (**d**) from DRG neurons treated as indicated. **e**, **f** Boltzmann fits for normalized conductance, G/Gmax, voltage relations for voltage dependent activation (**e**) and inactivation (**f**) of sensory neurons treated as indicated. V_1/2_ values for activation and inactivation are presented in Table 1. Asterisk indicate statistical significance compared with cells treated with 0.1% DMSO (*P < 0.05, unpaired two-tailed Student’s t test, *n* = 16–19 per condition)

Since sodium ion is a critical component in the generation of action potentials, modulating neuronal excitability and propagating nociceptive signaling, we assessed the possible action of 1-*O*-acetylgeopyxin A on Na^+^ currents by whole cell voltage-clamp electrophysiology (protocols illustrated in Fig. 3a). Typical families of sodium currents from DRG neurons treated with DMSO (*n* = 16) or 1-*O*-acetylgeopyxin A (*n* = 19) are shown in Fig. 3b. Overnight treatment with 1-*O*-acetylgeopyxin A (20 μM) inhibited total Na^+^ current density with ~ 47% decrease in peak current density (Fig. 3c, d); data displayed is normalized by cell capacitance. We next investigated the effect of 1-*O*-acetylgeopyxin A on the biophysical properties of voltage-dependence activation and inactivation of DRG Na^+^ currents. Steady-state activation (Fig. 3e) and inactivation properties (Fig. 3f) of sodium currents were affected. There was a depolarizing shift of ∼5 mV in the V_1/2_ with 1-*O*-acetylgeopyxin A (Table 1). Likewise, a depolarizing shift of ∼7 mV in the V_1/2_ of steady-state inactivation (Table 1), was reported; these data shows the inhibitory function of 1-*O*-acetylgeopyxin A on Na^+^ channels.

### TTX-sensitive sodium currents in DRG sensory neurons are reduced by 1-*O*-acetylgeopyxin A

**Fig. 4:**
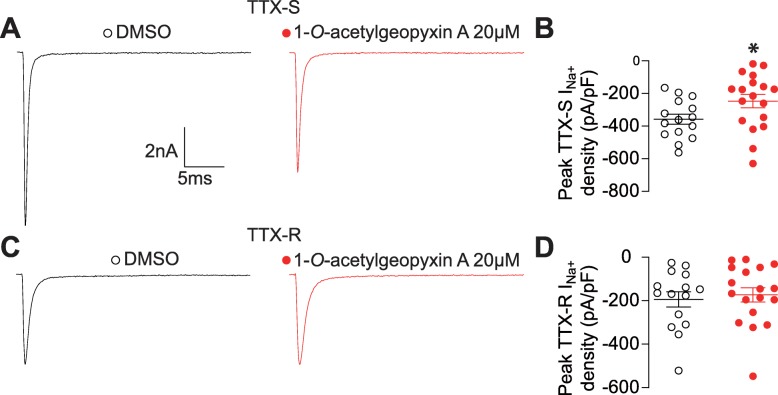
1-*O*-acetylgeopyxin A inhibits TTX-sensitive Na + currents in dorsal root ganglion (DRG) neurons. **a**,**c** Representative traces of TTX-sensitive (**a**) and TTX-resistant (**b**) Na^+^ currents from DRG sensory neurons treated with 0.1% DMSO (control) or 20 μM 1-*O*-acetylgeopyxin A. **b**, **d** Summary (± SEM) of the peak TTX-sensitive (**b**) or TTX-resistant (**d**) Na^+^ current density (mean ± SEM) from DRG neurons treated as indicated. TTX-sensitive and TTX-resistant fractions were calculated as described in the Methods section. Asterisk indicate statistical significance compared with cells treated with 0.1% DMSO (*P < 0.05, unpaired two-tailed Student’s t test, *n* = 15–18 per condition, Mann−Whitney test)

Na^+^ channels can be classified according to their sensitivity (NaV1.7) or resistance (NaV1.8 and NaV1.9) to tetrodotoxin (TTX) in DRG sensory neurons [20]. TTX-sensitive (TTX-S) currents activate at low thresholds, are fast-inactivating, shape the action potential and are required for initial depolarization [20]. As distinctive inactivation kinetics distinguish TTX-resistant (TTX-R) from TTX-S Na^+^ channels, a fast-inactivation protocol (see Methods) was used to electrically isolate TTX-R (current available following a − 40 mV prepulse) from total current (current left after a − 120 mV prepulse), as previously described [21]. DRG neurons were treated overnight with 20 μM of 1-*O*-acetylgeopyxin A or control (0.1% DMSO) as indicated, subsequently TTX-R and TTX-S Na^+^ currents were recorded and isolated. When compared to control, 1-*O*-acetylgeopyxin A significantly inhibits TTX-S Na^+^ currents (~ 31%) (Fig. 4a, b). Based on different properties of the DRG TTX-S and TTX-R Na^+^ currents, TTX-R currents were estimated by a 200 ms test pulse to 10 mV following 1000 ms prepulse at − 40 mV to inactivate the TTX-S component. 1-*O*-acetylgeoyxin A did not significantly inhibit TTX-R currents (Fig. 4c, d). Thereby, we conclude that 1-*O*-acetylgeopyxin A effects TTX-S Na^+^ currents in DRG neurons.

### 1-*O*-acetylgeopyxin a inhibits sensory neuron excitability

**Fig. 5:**
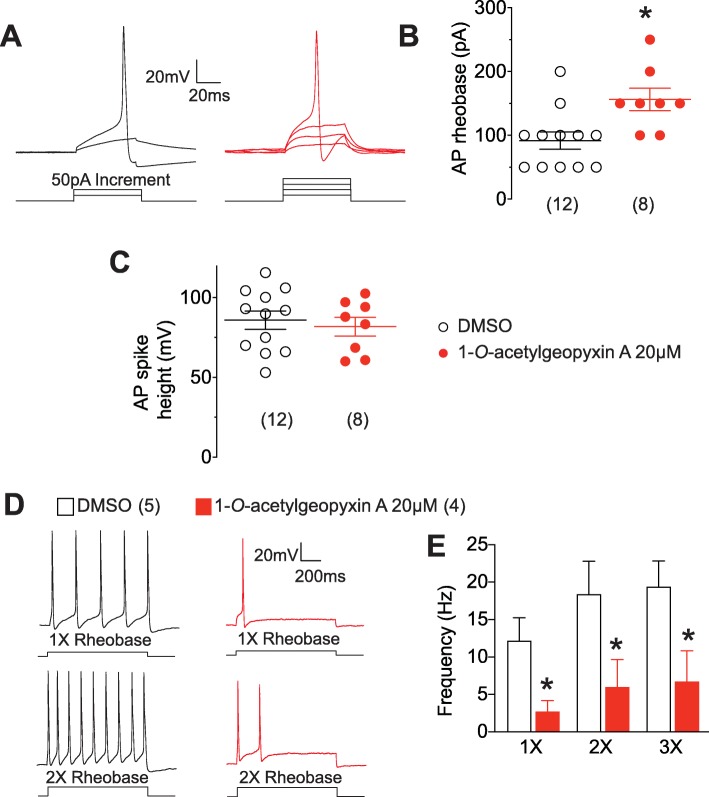
1-*O*-acetylgeopyxin A inhibits excitability in dorsal root ganglion (DRG) neurons. **a** Representative recordings in response to various steps of depolarizing current to measure rheobase (i.e. current required for eliciting the first action potential (AP)) in sensory neurons incubated with DMSO or 20 μM 1-*O*-acetylgeopyxin A. Summary of the measured mean (± SEM) rheobase (**b**) and AP spike height (in millivolts, mV) (**c**) in indicated conditions (n = 8–12 as indicated; (*P = 0.0085, unpaired two-tailed Student’s t test). **d** Representative traces of action potentials displayed by DRG neurons in culture after incubation with DMSO or 20 μM 1-*O*-acetylgeopyxin A in response to current injections of 1x or 2x rheobase. **e** Bar graphs of mean frequency of APs in response to current injections of 1x, 2x, or 3x rheobase. (*P < 0.05, one-way ANOVA with Dunnett’s post hoc test)

With effects on both voltage-gated sodium and calcium channels – gating of which is inextricably linked to neuronal excitability, we next assessed potential effects of expect 1-*O*-acetylgeopyxin A excitability properties of sensory neurons. Consistent with the decrease in total and TTX-S Na^+^ currents noted above, 1-*O*-acetylgeopyxin A application in sensory neurons resulted in decreased action potential (AP) frequency [12.2 ± 3.0 for DMSO control (*n* = 5) vs. 2.8 ± 1.4 for 1-*O*-acetylgeopyxin A (*n* = 4)] (Fig. 5a, b); increased rheobase [91.7 ± 13.5 for DMSO control (*n* = 12) vs. 156.3 ± 17.5 for 1-*O*-acetylgeopyxin A (*n* = 8)], the current required to initiate an action potential (Fig. 5c, d); but no change in AP spike height [85.8 ± 5.6 for DMSO control (n = 12) vs. 81.8 ± 5.9 for 1-*O*-acetylgeopyxin A (n = 8)] (Fig. 5e). Spontaneous activity was not observed in any of the 1-*O*-acetylgeopyxinA-treated DRGs (*n* = 13; data not shown). Together, these observations demonstrate that 1-*O*-acetylgeopyxin A inhibits sensory neuron excitability.

### HIV-induced sensory neuropathy is alleviated by treatment with 1-*O*-acetylgeopyxin a

**Fig. 6:**
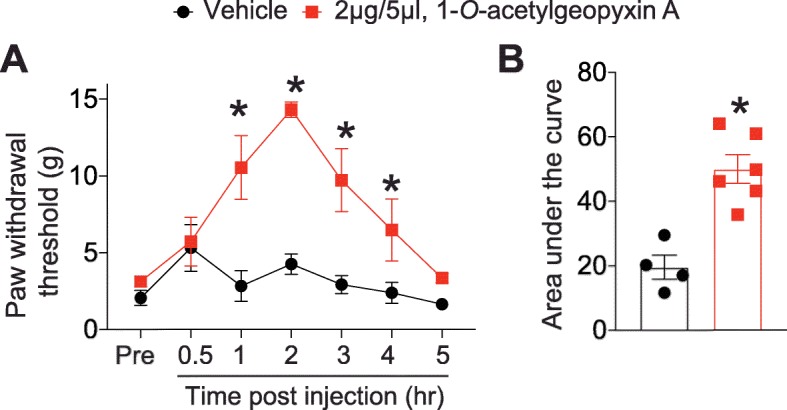
1-*O*-acetylgeopyxin A reduces gp120-induced mechanical allodynia. **a** Paw withdrawal threshold of adult male rats (*n* = 4-6) was measured 15 days after three intrathecal injections of gp120. Rats were treated with (intrathecally (i.th.) via catheter) saline (vehicle) or 1-*O*-acetylgeopyxin A (2 μg/5 μL) as indicated. Asterisks indicate statistical significance compared with saline treatment (*p < 0.05; two-way ANOVA with a Student− Neuman−Kuels post hoc test). **b** Area under the curve was derived as indicated above using GraphPad Prism. Statistical significance is indicated by asterisks (*p < 0.05, Mann−Whitney test) in comparison to vehicle-treated rats. Experimenter was blinded to the treatment condition.

Since voltage-gated Ca^2+^ and Na^+^ channels have been shown to play an important role in neuropathic pain [22–26], we then explored the potential of 1-*O*-acetylgeopyxin A in alleviating neuropathic pain in an experimental model. We selected the HIV-induced sensory neuropathy model as both calcium and voltage-gated channels have been linked to pain in this model. To test this, rats were administered with three intrathecal (i.th.) injections of the HIV-1 envelope glycoprotein (gp120) which resulted in the development of mechanical allodynia (Fig. 6a), consistent with previous reports [27, 28]. I.th injection of 1-*O*-acetylgeopyxin A (2 μg/5 μL) reversed mechanical allodynia 1–2 h post-injection and lasted for an 2 additional hours (Fig. 5a). This reversal is supported by a significant increase of the area under the curve between 1-*O*-acetylgeopyxin A treated animals the vehicle (saline) treated animals (Fig. 6b). Therefore, our results indicate that 1-*O*-acetylgeopyxin A has antinociceptive potential for HIV-induced sensory neuropathy.

## Discussion

*Ent-*kauranes represent an important group of tetracyclic diterpene natural products and their structures are constituted by a perhydrophenantrene unit fused with a cyclopentane unit formed by a bridge of two carbons between C-8 and C-13 [5]. Diverse biological activities have been reported for *ent-*kaurane diterpenoids [6–15]. However, it has not yet been studied if these molecules have an antinociceptive effect. Therefore, the purpose of this work was to identify if *ent-*kauranes or their analogs have a therapeutic potential for pain relief.

In this context, in the present work we report that 1-*O*-acetylgeopyxin A, a derivative of the new *ent-*kaurane diterpenoid geopyxin A, inhibits voltage-gated Ca^2+^ channels (Fig. 2) and voltage-gated Na^+^ channels (Fig. 3), mediating the inhibition preferentially through the TTX-sensitive Na + channels (Fig. 4). Notably, 1-*O*-acetylgeopyxin A reversed mechanical allodynia on HIV-induced rodent model of sensory neuropathy (Fig. 5). The block of Ca^2+^ and tetrodotoxin-sensitive Na^+^ channels supported the antinociceptive effect of 1-*O*-acetylgeopyxin A (Fig. 5). Nevertheless, it is important to determine the member(s) of voltage-gated Ca^2+^ channels underlying this inhibition, since rat DRG neurons express high-voltage activated (HVA; L-type (CaV1), P/Q-type (CaV2.1), N-type (CaV2.2), and R type (CaV2.3)) and low voltage-activated (LVA; T-type (CaV3)) calcium channels. While not knowing which Ca^2+^ channel is targeted by 1-*O*-acetylgeopyxin A is a limitation of this study, dual or multi-target antagonists may have a better efficacy profile. Along these lines, it was recently reported that a derivatized version of a cationic N-type calcium channel blocker inhibited CaV2.2 (N-type) calcium channels as well as both tetrodotoxin-sensitive and tetrodotoxin-resistant voltage-gated sodium channels and was highly effective in producing long-lasting analgesia in mouse models of surgical and inflammatory pain [29]. Further, the molecular action of gabapentinoids may involve the calcium channel auxiliary protein α2δ-1’s action on CaV2.2 [30], thrombospondin [31], or N-methyl-D-aspartate receptors [32], highlighting the potential of multi-target engagement to elicit analgesia.

Voltage-gated Ca^2+^ channels are critical targets in nociceptive transmission [24–26]. HVA Ca^2+^ channels, primarily the CaV2 family, are expressed in presynaptic terminals on the spinal cord dorsal horn and play an important role in neurotransmitter release and neuronal excitability [33–35], while LVA Ca^2+^ channels, in addition to participating in low-threshold exocytosis [36], are also involved in shaping action potentials, regulating neuronal firing patterns, lowering action potential thresholds, promoting burst firing, oscillatory behavior and enhancing synaptic excitation [37]. All these channels have been associated with painful phenotypes. For example, during neuropathic pain, an enhanced expression of the HVA calcium channels in DRG and presynaptic terminals has been reported [38, 39]. It is well known that the increased expression of these channels in the plasma membrane is mediated by their association with the auxiliary subunit α2δ-1, which has also been found to increase in neuropathic pain [40–42]. Likewise, LVA Ca^2+^ channel expression is augmented in chronic pain [25, 43, 44]. Thus, in animal models of neuropathic pain, enhanced expression of these channels results in an increase in the maximum Ca^2+^ current density with the consequent increase in neuronal excitability [25, 39]. Therefore, blocking Ca^2+^ channels with 1-*O*-acetylgeopyxin A (Fig. 2), resulted in the relief of mechanical allodynia (Fig. 5), which is consistent with previous reports using ziconotide [45], a N-type Ca^2+^ channel blocker and TTA-P2 [46], a T-type Ca^2+^ channel blocker to relieve pain [45, 46].

Voltage-gated sodium channels are also key components in the nociceptive pathway due to their electrical properties and roles in propagating electrical signaling. NaV1.7, NaV1.8, and NaV1.9 subtypes of NaV channels are preferentially found in the peripheral nervous system and are known to be crucial in the conduction of nociceptive stimuli [20]. These channels can be classified according to their sensitivity (NaV1.7) and resistance (NaV1.8 and NaV1.9) to TTX [20] in sensory neurons. Nav1.8 is preferentially expressed in large and medium DRG neurons [47] and NaV1.9 in small-diameter DRG neurons [48], while NaV1.7 is expressed in small- and medium-diameter DRG neurons (and rarely in large-diameter neurons). NaV1.7 produces a rapidly activating and inactivating TTX-sensitive current [49]. This channel is well suited to low-frequency firing in nociceptive C-fibers due to its slow repriming nature [49] and is considered a threshold channel due to its ability to boost subthreshold stimuli, thereby increase action potential firing frequency [50]. The TTX-R current produced by NaV1.8 acts as the main contributor to the upstroke of action potentials [51]. NaV1.9 can be activated at voltages close to resting membrane potential and generates a persistent TTX-R current that has very slow gating kinetics. Therefore, this channel acts as a modulator of membrane excitability through persistent inward currents [48]. Several rodent studies have demonstrated the importance of these channels in pain sensation. Mice lacking NaV1.7 in NaV1.8 positive nociceptors displayed insensitivity to painful stimuli [52]. Similarly, deletion of NaV1.8 in sensory neurons reduces the sensitivity to noxious mechanical [51] and NaV1.9 knockout mice do not develop thermal hyperalgesia after complete Freund’s adjuvant or carrageenan injection [53]. Our data shows that 1-*O*-acetylgeopyxin A did not affect NaV1.8 and Nav1.9 currents (Fig. 4). Hence, 1-*O*-acetylgeopyxin A appears to be involved in inhibition of pan-TTX-S Na^+^ currents. Overall, the inhibition of total Na^+^ currents in sensory neurons (Fig. 3), likely via its action on NaV1.7 (Fig. 4) as well as an inhibition of sensory neuron excitability (Fig. 5) collectively contributes to inhibition of nociceptive behavior (Fig. 6).

Thus, our findings support the use of 1-*O*-acetylgeopyxin A as a lead compound, positioning its structure for future modifications and optimizations for the eventual production of more effective antinociceptive drugs.

## Methods

### Animals

Adult Sprague−Dawley rats (females, 225 − 250 g; Harlan Laboratories) were kept in light-controlled (12 h light/12 h dark cycle; lights on 07:00–19:00) and temperature-controlled (23 ± 2 °C) rooms with access to rodent chow and water as needed. The University of Arizona’s College of Medicine Institutional Animal Care and Use Committee (IACUC) sanctioned all experiments. All experiments were performed per guidelines recommended published by National Institutes of Health Guide for Care and Use of Laboratory Animals and adhered to International Association for the Study of Pain ethical guidelines. For the behavioral experiments, rats were randomly assigned to control or treatment conditions. Animals were initially housed three/cage and singly after the intrathecal cannulation on a 12 h dark−light cycle with ad libitum food/water. Experimenters performing the behaviors were kept blinded to the experimental treatment conditions.

### Materials

All utilized chemicals and reagents were purchased from Sigma (St. Louis, MO) unless otherwise stated. The natural products and their derivatives used in this study, geopyxin A, 1-*O*-acetylmethylgeopyxin A, methylgeopyxin A, 1-*O*-acetylgeopyxin A and geopyxin C were obtained as described previously [5].

### Preparation of acutely dissociated dorsal root ganglion neurons

Dorsal root ganglia from all spinal levels were cultured using methods as described previously [54, 55]. Dissociated DRG neurons were subsequently plated onto 12- or 15-mm poly-D-lysine and laminin-coated coverslips and cultured for up to 48 h in media consisting of DMEM (1% penicillin/streptomycin sulfate from 10,000 μg/mL stock, 10% fetal bovine serum (Hyclone)), and 30 ng/mL nerve growth factor).

### Calcium imaging in acutely dissociated rat dorsal root ganglia neurons

Calcium imaging was done as described previously [17]. Since the K^+^ concentration inside the cell is 140 mM, Cl^−^ is 10 mM, and Na^+^ is 15 mM, then at room temperature (25 degree Celsius), using the Nernst equation, a trigger with 40 mM KCl will bring the membrane voltage to − 32.2 mV, which activates mostly LVA calcium channels. When challenged with 90 mM KCl, the membrane voltage changes to − 11.4 mV, which activates HVA; and at this potential, most LVA is already inactivated (for example, see [56] or [57]). Dorsal root ganglion neurons were bathed for 30 min at 37 °C with a concentration of 3 μM Fura-2 AM (Cat# F1221, Thermo Fisher, stock solution prepared at 1 mM in DMSO, 0.02% pluronic acid, Cat#P-3000MP, Life Technologies) to survey changes in intracellular calcium([Ca^2+^]c) as described before [58]. The changes in [Ca^2+^]c changes were examined with a ratio of F340/ F380, calculated after subtracting background from both channels.

### Whole-cell electrophysiological recordings of calcium currents in acutely dissociated DRG neurons

Recordings of total calcium currents were obtained using recording solutions and protocols (also illustrated in the figures) described earlier [18]. The internal solution consisted of (in mM): 150 CsCl_2_, 10 HEPES, 5 Mg-ATP, and 5 BAPTA (pH 7.3, mOsm/L = 290–310) and external solution contained (in mM): 110 NMDG, 10 BaCl_2_, 30 TEA-Cl, 10 HEPES, 10 glucose and 10 μM HEPES (pH 7.3, mosM/L = 310–315). The neurons were subjected to current−density (I − V) and activation/inactivation voltage protocols as previously described and shown in the figures [18].

### Whole-cell electrophysiological recordings of sodium currents in acutely dissociated DRG neurons

Recordings were obtained from acutely dissociated DRG neurons as described by us before [55, 59, 60]. The internal solution consisted of (in mM): 140 CsF, 10 NaCl, 1.1Cs-EGTA, and 15 HEPES (pH 7.3, mOsm/L = 290–310) and external solution contained (in mM): 140 NaCl, 30 tetraethylammonium chloride, 10 D-glucose, 3 KCl, 1 CaCl2, 0.5 CdCl_2_, 1 MgCl_2_, and 10 HEPES (pH 7.3, mosM/L = 310–315). The neurons were subjected to current−density (I− V) and activation/inactivation voltage protocols as previously described and shown in the figures [18, 21]. Because of differential inactivation kinetics of TTX-R and TTX-S channels, the fast inactivation protocol permitted separation of electrically isolated TTX-R (current available following a − 40 mV prepulse) from the total current (current left after a − 120 mV prepulse), to obtain TTX-S currents, as previously reported [21]. TTX-R current was estimated by a 200 ms test pulse to 10 mV following 1000 ms prepulse at − 40 mV to inactivate the TTX-S component [61]. To test for TTX-resistant currents, I − V protocol was conducted after 5 min of bathing in a concentration of 1 μM TTX. Pipettes with 1–3 MΩ resistance were used for all recordings.

### Whole-cell patch recording of spontaneous and evoked action potential in acutely dissociated DRG neurons

Current-clamp recordings were performed as described previously [62]. Briefly, the internal solution contained (in mM): 137 KCl, 10 NaCl, 1 MgCl_2_, 1 EGTA, and 10 HEPES adjusted to pH 7.3 with KOH. The external solution contained (in mM): 154 NaCl, 5.6 KCl, 2 CaCl_2_, 1 MgCl_2_, 10 Glucose and 8 HEPES adjusted to pH 7.4 with NaOH. The DRG neurons with a resting potential more hyperpolarized than − 40 m V, stable baseline recordings, and evoked spikes that overshot 0 mV were used for experiments and analysis. To measure DRG excitability, first, a series of current steps that were 500-millisecond in duration and of 10-pA steps from − 50 pA were used to determine the rheobase; second, neurons were held at resting potentials and injected with a series of 1-s steps of depolarizing current with an amplitude of 1 × rheobase, 2 × rheobase and 3 × rheobase. The DRG neurons were then held at 0 pA to record spontaneous action potential for 5 min. Bridge balance was compensated to above 60% for the recorded DRG neurons. All recordings were made at room temperature.

### Intrathecal catheterization

For drug administration via intrathecal (i.t.) route, rats were implanted with catheters as described by Yaksh and Rudy [63].

### Assessment of mechanical allodynia

Allodynia was tested as described previously [17]. Data was analyzed as reported by Chaplan et al. [64] using Dixon’s nonparametric method.

### HIV-induced sensory neuropathy

Mechanical allodynia is produced by i.th. injection of human immunodeficiency virus-1 (HIV-1) envelope glycoprotein, gp120 [28]. The compounds were assessed for their ability to affect mechanical allodynia at 10–14 days after the first injection of gp120.

### Statistical analyses

All values represent the mean ± SEM. Data sets were all tested with a D’agostino − Pearson test for normality (Graphpad Prism 8 Software). Following the result of the normality test, statistical significance was tested using the appropriate parametric or nonparametric Student’s t test or analysis of variance (ANOVA), after which we performed post hoc comparisons (Tukey). Von Frey behavioral data sets were analyzed by two-way ANOVA (Tukey posthoc test). Statistical significance was inferred for all *p* ≤ 0.05. GraphPad Prism 8 was used for all graphs. No data points were excluded in our studies.

## Data Availability

Please contact author for data requests.

## Abbreviations

KCl: Potassium chloride
DRG: Dorsal root ganglia
TTX: Tetrodotoxin
TTX-S: Tetrodotoxin-sensitive
TTX-R: Tetrodotoxin-resistant
LVA: Low voltage-activated
HVA: High voltage-activated
V_1/2_: Half-maximal activation
*k*: Slope
HIV gp120: Human immunodeficiency virus envelope glycoprotein of 120 KDa
